# Fungal Interactions and Host Tree Preferences in the Spruce Bark Beetle *Ips typographus*

**DOI:** 10.3389/fmicb.2021.695167

**Published:** 2021-06-04

**Authors:** Sifat Munim Tanin, Dineshkumar Kandasamy, Paal Krokene

**Affiliations:** ^1^Division of Biotechnology and Plant Health, Norwegian Institute of Bioeconomy Research, Ås, Norway; ^2^Faculty of Environmental Sciences and Natural Resource Management, Norwegian University of Life Sciences, Ås, Norway; ^3^Chair of Forest Entomology and Protection, University of Freiburg, Freiburg, Germany; ^4^Department of Biochemistry, Max Planck Institute for Chemical Ecology, Jena, Germany

**Keywords:** bioassays, *Endoconidiophora rufipennis*, *Grosmannia penicillata*, host choice, *Leptographium abietinum*, *Endoconidiophora polonica*

## Abstract

The spruce bark beetle *Ips typographus* is the most damaging pest in European spruce forests and has caused great ecological and economic disturbances in recent years. Although native to Eurasia, *I. typographus* has been intercepted more than 200 times in North America and could establish there as an exotic pest if it can find suitable host trees. Using *in vitro* bioassays, we compared the preference of *I. typographus* for its coevolved historical host Norway spruce (*Picea abies*) and two non-coevolved (naïve) North American hosts: black spruce (*Picea mariana*) and white spruce (*Picea glauca*). Additionally, we tested how *I. typographus* responded to its own fungal associates (conspecific fungi) and to fungi vectored by the North American spruce beetle *Dendroctonus rufipennis* (allospecific fungi). All tested fungi were grown on both historical and naïve host bark media. In a four-choice Petri dish bioassay, *I. typographus* readily tunneled into bark medium from each of the three spruce species and showed no preference for the historical host over the naïve hosts. Additionally, the beetles showed a clear preference for bark media colonized by fungi and made longer tunnels in fungus-colonized media compared to fungus-free media. The preference for fungus-colonized media did not depend on whether the medium was colonized by conspecific or allospecific fungi. Furthermore, olfactometer bioassays demonstrated that beetles were strongly attracted toward volatiles emitted by both con- and allospecific fungi. Collectively, these results suggest that *I. typographus* could thrive in evolutionary naïve spruce hosts if it becomes established in North America. Also, *I. typographus* could probably form and maintain new associations with local allospecific fungi that might increase beetle fitness in naïve host trees.

## Introduction

Microbial symbionts of invasive insect herbivores often promote successful establishment and development in host plants in novel habitats (Wingfield et al., 2010, 2016; Hulcr and Dunn, 2011; Frago et al., 2012). Co-invading microbes may help their insect vector by outcompeting native microbes that would reduce insect fitness in novel habitats or by inhibiting pathogens, competitors, and predators of the invading insect (Prenter et al., 2004). Alternatively, invasive insects could form novel associations with native microbes that promote their establishment in naïve habitats (Himler et al., 2011; Jiggins and Hurst, 2011; Zhao et al., 2013). Microbes can thus facilitate rapid adaptation of invasive insects to local conditions and increase the damage they cause in new habitats.

Many insects have invaded novel forest ecosystems worldwide and some of these species cause great ecological and economic disturbances (Aukema et al., 2010). Wood products and wood packing materials used in global trade are important pathways for the spread of bark- and wood-boring insects (Økland et al., 2011). Bark beetles in the sub-family Scolytinae were intercepted 6,825 times near United States ports of entry between 1985 and 2000 (Haack, 2001), and one of the most frequently detected species is the Eurasian spruce bark beetle, *Ips typographus* L., with 465 interceptions up until 2008 (Liebhold et al., 2017). *Ips typographus* is a tree-killing bark beetle that primarily colonizes Norway spruce [*Picea abies* (L.) Karst.], the economically most valuable forest tree species in Europe (Schroeder and Cocoş, 2018). Outbreaks of *I. typographus* and other tree-killing bark beetles have killed hundreds of millions of trees in recent years, with devastating economic impacts in commercial production forests (Raffa et al., 2008; Hlásny et al., 2019).

Conifer trees have evolved multiple mechanisms to defend themselves against bark beetle attack, including the production of chemical defenses such as terpenoid-rich oleoresin and phenolics (Keeling and Bohlmann, 2006; Celedon and Bohlmann, 2019). To avoid these co-evolved defenses, bark beetles usually colonize weak or dying trees with impaired defenses, such as storm- and drought-stressed trees (Marini et al., 2017). However, when their populations are high a few tree-killing species can also overcome the resistance of relatively healthy trees and initiate landscape-scale outbreaks (Raffa et al., 2008). These tree-killing species tend to be assisted by different assemblages of phytopathogenic ophiostomatoid fungi in the genera *Endoconidiophora*, *Ophiostoma*, and *Grosmannia* (Kirisits, 2007; Krokene, 2015). The exact species composition of these fungal assemblages usually varies over time and space (Linnakoski et al., 2012, 2016; Chang et al., 2019).

Terpene-rich resins and phenolics in the bark are toxic to different life stages of bark beetles and to many of their fungal associates (Gershenzon and Dudareva, 2007; Krokene, 2015; Hammerbacher et al., 2019). Phytopathogenicity and ability to metabolize the trees’ chemical defenses are important fungal adaptations to survive in the highly defensive environment of a healthy conifer tree (DiGuistini et al., 2011; Six and Wingfield, 2011). Two of the most virulent fungal symbionts of *I. typographus*, *Grosmannia penicillata* (Grosmann) Goid. and *Endoconidiophora polonica* (Siemaszko) de Beer et al. (2014), produce large necrotic lesions in the inner bark (phloem) of Norway spruce and can effectively metabolize spruce chemical defenses (Krokene and Solheim, 1998, 2001; Zhao et al., 2019). These fungi may thus exhaust and overcome the tree’s chemical defenses and ultimately help their beetle vectors breed in the phloem (Lieutier et al., 2009). Fungal symbionts may also provide nutritional benefits to bark beetle larvae by increasing nitrogen levels in the phloem (Bleiker and Six, 2007; Six and Elser, 2019). Recently, volatiles released by key fungal symbionts have also been suggested to serve as recognition cues that help newly eclosed adult beetles locate and pick up beneficial fungi from their brood tree and avoid poorly performing fungi (Kandasamy et al., 2019; Zhao et al., 2019).

If tree-killing bark beetles are introduced outside their natural range, they are usually exposed to novel host trees that lack co-evolved defenses. Such evolutionary naïve tree species are often favorable hosts as they have little resistance against invasive insects and their symbionts (Cudmore et al., 2010; Raffa et al., 2013; Sun et al., 2013). In North America, several spruce species are potential hosts for *I. typographus*, including white spruce [*Picea glauca* (Moench) Voss] and black spruce [*Picea mariana* (Mill.) B.S.P.]. These species are frequently colonized by the North American spruce beetle *Dendroctonus rufipennis* Kirby, a native bark beetle vectoring ophiostomatoid fungi, such as *Leptographium abietinum* (Peck) M.J. Wingf. and *Endoconidiophora rufipennis* (M.J. Wingf., T.C. Harr. and H. Solheim) de Beer et al. (2014), that are moderately virulent to their host trees (Solheim and Safranyik, 1997). *Leptographium abietinum* appears to benefit its bark beetle vector by providing vital nutrients, metabolizing toxic host terpenes, and protecting beetles from an entomopathogenic fungus (Davis et al., 2019). *Ips typographus* has previously been shown to breed successfully in several North American spruce species (Økland et al., 2011; Flø et al., 2018) and if introduced in North America it will probably be exposed to the fungal symbionts of *D. rufipennis*. However, it is not clear how *I. typographus* responds behaviorally to its own fungi and fungi associated with *D. rufipennis*, or how *I. typographus*’ fungal symbionts perform in evolutionary naïve spruce hosts.

One objective of this study was to test the pathogenicity of fungi typically associated with *I. typographus* (hereafter referred to as conspecific fungi) and *D. rufipennis* (hereafter referred to as allospecific fungi) to three spruce species: the historical host Norway spruce and the evolutionary naïve white and black spruce. As fungal volatiles can be attractive to bark beetles, we also examined how volatile profiles of conspecific and allospecific fungi influence their interactions with *I. typographus*. Lastly, we evaluated how different spruce species affect host acceptance and tunneling of *I. typographus* in the presence and absence of con- and allospecific fungi.

## Materials and Methods

### Harvesting Spruce Trees

We harvested nine 55-year-old Norway spruce (*P. abies*), black spruce (*P. mariana*), and white spruce (*P. glauca*) trees growing near Prestebakke, Halden (58.99°N, 11.54°E). The 27 trees were felled on 22 April 2018 and transported to Ås the next day. From each log, we cut four 40–45 cm long bolts and sealed the cut ends with melted paraffin wax to reduce desiccation and evaporation of resin and other chemicals. One bolt from each tree was used for fungal pathogenicity tests, two bolts were used to rear beetles for the bark beetle bioassays, and one bolt was used to harvest bark to prepare spruce bark media for bioassays.

### Fungal Pathogenicity Tests in Spruce Bolts

To test fungal pathogenicity in Norway spruce, black spruce and white spruce, we used two fungal species that are common associates of *I. typographus* (conspecific fungi) and two species that are associates of *D. rufipennis* (Solheim and Safranyik, 1997; allospecific fungi; Table 1). To test how con- and allospecific fungi performed in the three spruce species, we inoculated two isolates of each fungus into a single cut bolt from each of the 27 felled spruce trees. The bolts were stored at 4°C from 7 May to 14 August, when they were taken to an insectarium (a large shaded room with netted walls, providing close to ambient temperature, humidity, and wind flow). The bolts were allowed to warm up to ambient temperature before they were inoculated with fungi on 16 August.

**Table 1:** Species of bark beetle-associated fungi inoculated in cut bolts of Norway spruce, black spruce, and white spruce.

Fungus	Isolate number	Beetle vector	Geographical range
*Endoconidiophora polonica*	1993–208/115^∗^^EP1^	*Ips typographus*	Europe
	1997–770/9^EP2^		
*Grosmannia penicillata*	2006–209/44/2^∗^^GP1^		
	1980–91/54^GP2^		
*Endoconidiophora rufipennnis*	1992–633/262/9^ER1^	*Dendroctonus rufipennis*	North America
	1993–403/463^∗^^ER2^		
*Leptographium abietinum*	1992–635/310/4^∗^^LA1^		
	1992–633/9/2^LA2^		

Isolate numbers refer to the culture collection of the Norwegian Institute of Bioeconomy Research.

**Figure 1:**
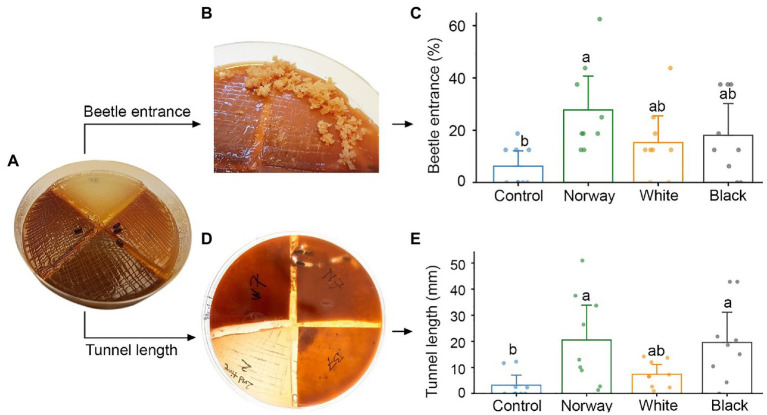
Testing adult *Ips typographus* host preferences in multi-species choice arenas with a quadrant of water agar medium (control) and quadrants of spruce bark agar medium made from Norway spruce, white spruce, and black spruce. **(A)** Four beetles were released in each arena. **(B,C)** The number of beetles entering different media was registered after 18 h. **(D,E)** Beetle tunneling length in different media was measured after 5 days. Bars show meanvalues with 95% CIs. Dots represent individual replicates (*n* = 9 individual trees per spruce species). Treatments with different letters (a, b) differed significantly (ANOVA with Tukey’s *post hoc* test).

∗ Isolate that was also used in beetle choice studies. Superscript number corresponds to the isolate numbers used in Figure 1.

Each spruce bolt was inoculated with all eight fungal isolates (Table 1) as well as mock controls. The bolts were inoculated by removing a plug of bark using a 5 mm cork borer, inserting inoculum into the hole, and gently putting the bark plug back in place, ensuring that most of the inoculum stayed inside the hole. Each bolt was inoculated twice with the mock control (a 5 mm plug of sterile malt agar: 2% agar, 1.4% malt) and once with a 5 mm plug of each fungal isolate growing on malt agar. The 10 inoculations per bolt were distributed among two bands encircling the bolt about 10 cm from each end. To prevent necrotic lesions extending from upper and lower inoculation from coalescing, inoculation sites were offset so that upper and lower inoculation sites never were situated right below each other. Around 3 weeks after inoculation (6 September 2018), we removed the outer bark over all inoculation sites and measured the full length of the necrotic lesions in the inner bark.

### Rearing Beetles for Bioassays

Two bolts from each of the 27 felled spruce trees were brought to a fresh clear-cut in Ås (59.65°N, 10.81°E) on 7 May 2018 and left there for 3 weeks to be colonized by *I. typographus*. The bolts were then collected and hung from the ceiling in the insectarium. Each bolt was covered in an emergence net attached to a large plastic funnel with a collection bottle beneath, to ensure that all beetles emerging from the bolt would be collected. Beetles used in the bioassays were taken from the collection bottles or from the bark that was peeled from the logs at the end of the rearing period. For later follow-up bioassays, we used additional beetles from naturally infested Norway spruce trees in Ås. On 27 June 2019, we cut several bolts from three windfelled Norway spruce trees that had been colonized by *I. typographus* in the same spring. The bolts were taken to the insectarium the same day and hung from the ceiling with collection funnels attached. All collected beetles were stored at 4°C for maximum 5 days before they were used in bioassays. To reduce any variability in beetle quality caused by host bark medium, we only used beetles that had developed in Norway spruce bolts in our bioassays. Before placing beetles in bioassay arenas, the sex of the beetles was determined using the characteristics described in Schlyter and Cederholm (1981).

### Preparation of Semi-Natural Spruce Bark Media

Semi-natural spruce bark media for bioassays were prepared as follows. We peeled the inner bark from one bolt per tree, placed the bark in individual plastic bags, and stored it at −20°C. Bark powder was later prepared by grinding pieces of inner bark to a fine powder in liquid nitrogen using a Retsch MM300 Mixer Mill (Retsch, Haag, Germany). The semi-natural spruce bark medium was prepared as described in Kandasamy et al. (2019): 7% finely ground bark powder was mixed with 4% agar and heat-sterilized at 121°C for 20 min. Approximately 25 ml medium was poured into Petri dishes (92 mm diameter). We prepared several Petri dishes with bark medium from each of the 27 spruce bolts (i.e., from each tree individual). Additionally, several control Petri dishes were made with water agar (4% agar).

### Preparation of Host Choice Arenas With Multiple or Single Spruce Species

When the bark medium had cooled down and solidified in the Petri dishes, we prepared two different kinds of host choice arenas: multi-species arenas to test beetle preferences for different spruce species, and single-species arenas to test if fungal colonization of bark media influenced beetle choice.

Multi-species arenas were made by combining quadrants of bark medium from Norway spruce, black spruce, and white spruce as well as quadrants of control water agar medium in new Petri dishes (Figure 1). Quadrants of bark medium from each of the 27 spruce trees were thus combined with quadrants of control medium from nine Petri dishes to make a total of 36 multi-species choice arenas. In this way, bark medium from each spruce tree was distributed among four choice arenas constituting four technical replicates of each true biological replicate (i.e., individual tree). A 2 mm wide strip of sterile agar was used to physically separate the four different medium types in each arena (Figure 1).

**Figure 2:**
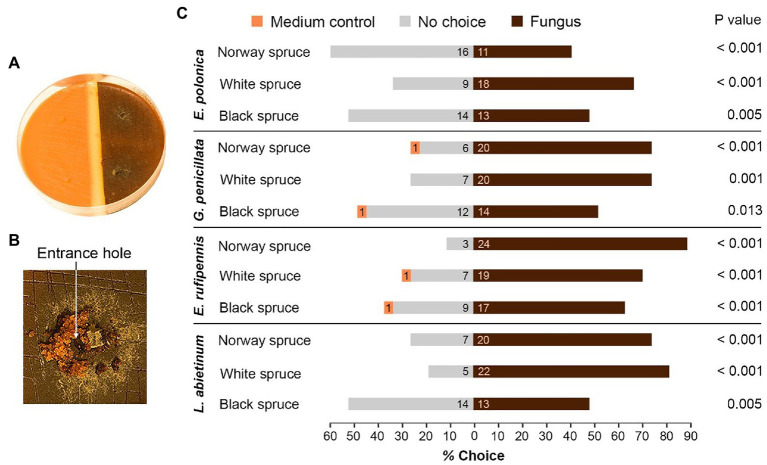
Host preferences of adult *I. typographus* in the presence of bark beetle-associated fungi. **(A)** Single-species choice arena containing semi-circles of bark agar medium made from Norway spruce, black spruce, or white spruce. The medium was either colonized by one of four fungal species 15 days earlier (right) or was left un-colonized (medium control, left). **(B)** Three beetles were released into each arena. Most of beetles entered fungus-colonized medium just beside the fungal inoculation plug (arrow). **(C)** The percentage of beetles that entered un-colonized medium (Medium control), fungus-colonized medium (Fungus) or remained on the surface (No choice) 48 h after introduction of beetles to the arenas (numbers inside the bars show total number of beetles that made their choice). *p* values show the outcome of Wilcoxon signed-rank tests comparing fungus and medium control for each combination of spruce species and fungal species (*n* = 9 individual trees per spruce species). E, *Endoconidiophora*; G, *Grosmannia*; and L, *Leptographium*.

Single-species choice arenas were made in a similar way by combining semi-circles of bark medium made from Norway spruce, black spruce, or white spruce trees. Each arena contained bark medium from an individual spruce tree that had either been colonized by fungus or was un-colonized (medium control). We first made eight Petri dishes from the bark medium prepared from each individual spruce tree, resulting in a total of 216 Petri dishes from all 27 trees. Four plates were inoculated with one of the four fungi listed in Table 1 and incubated for 15 days at 25°C. For these bioassays, we used only the most virulent isolate of each fungal species (see Table 1), in order to keep the number of treatment combinations at a manageable level. The remaining four Petri dishes from each spruce tree were left un-colonized. Choice arenas were then made by combining semi-circles of spruce medium from the same tree individual with or without fungal colonization and sealing the gap between the semi-circles with a 2 mm wide strip of sterile agar (Figure 2). This provided two technical replicates for each combination of individual spruce tree and fungal species.

### Host Choice Arenas With Multiple Spruce Species: Testing Beetle Host Preferences

Beetle preferences for different spruce species were tested using the multi-species choice arenas with bark medium from Norway spruce, black spruce, and white spruce, as well as agar control medium. We released four vigorous beetles into the center of each arena, attempting to keep an equal ratio of males and females. The Petri dish lid was then closed and sealed using parafilm to prevent the beetles from escaping. The arenas were left in the dark at 20°C for 18 h, before beetle host choice was evaluated non-intrusively by counting the number of beetle entrance holes in each arena quadrant. Around 5 days after the beetles were let into the arenas, we turned the arenas upside down and photographed any beetle tunnels made in the media. Total tunnel length per media type was quantified from the images using the image processing software ImageJ (Schneider et al., 2012).

### Choice Arenas With Single Spruce Species: Testing if Fungi Influence Beetle Host Preferences

To test if fungal colonization influence beetle host choice, we used the single-species choice arenas with fungus-colonized and un-colonized bark medium from each spruce species. Thus, the beetles had a choice between bark medium only and the same bark medium colonized by one of the four fungal species listed in Table 1. Three vigorous beetles were released into each arena, including at least one beetle of each sex. The arenas were then closed, sealed with parafilm, and left in the dark 20°C. After 48 h, the choice of the beetles and their tunnel length were non-intrusively evaluated using the method described for multiple-species arenas.

### Trap Bioassays: Testing the Attraction of Beetles Toward Fungal Volatiles

**Figure 3:**
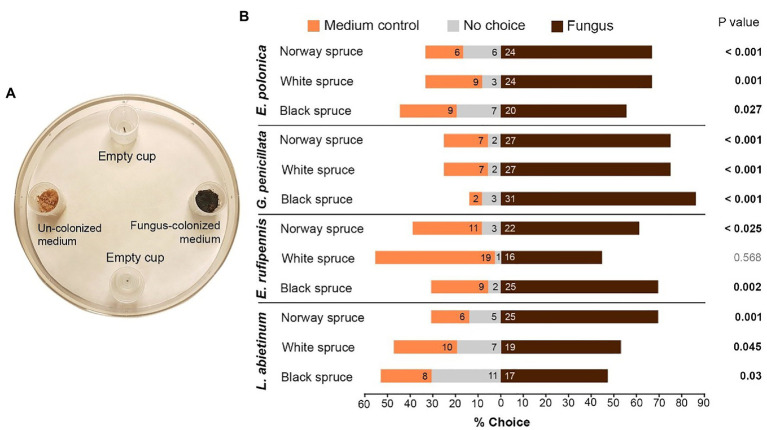
**(A)** Trap bioassays to test attraction of *I. typographus* toward traps baited with fungus-colonized spruce bark medium (Fungus) or un-colonized medium (Medium control). Empty traps served as a negative control. Two beetles were released into each test arena. **(B)** The percentage of beetles that entered the different traps or remained in the test arena (numbers inside the bars show actual beetle numbers). *p* values indicate the outcome of Wilcoxon signed-rank tests comparing fungus and medium control for each combination of spruce species and fungal species (*n* = 18; two independent tests using nine individual trees per spruce species). E, *Endoconidiophora*; G, *Grosmannia*; and L, *Leptographium*.

In a final series of bioassays, we tested if beetles can choose bark medium colonized by fungi based on olfaction, using a trap bioassay design described in Kandasamy et al. (2019; Figure 3). Four semi-transparent traps made from plastic cups (Ø 18 mm) were attached to the base of a large Petri dish (Ø 130 mm) arena using push-pins. With their opening facing upward, the traps were attached at an equal distance from each other and with 20 mm from the center of the trap to the wall of the Petri dish. To enable the beetles to enter the traps, four equidistant holes (Ø 4 mm) were made around the perimeter of each cup, 9 mm above the bottom of the Petri dish. We also made eight small holes (Ø 1 mm) in the Petri dish wall to allow air flow. The bottom of the Petri dish was covered with filter paper to provide a rough surface for walking beetles. Before the start of bioassays, two traps in each Petri dish arena were loaded with different odor sources. One trap was loaded with a bark medium plug (Ø 10 mm) from Norway spruce, black spruce, or white spruce that had been colonized by one of the fungal isolates listed in Table 1, following the procedure described above (“Preparation of host choice arenas with multiple or single spruce species”). Another trap at the opposite side of the arena was loaded with un-colonized bark medium from the same spruce tree, and the remaining two traps were left empty as blank controls (Figure 3). Two vigorous beetles were released in each arena and the Petri dish was closed, using a rubber band that sealed the space between the lid and the lower part of the Petri dish. To test for differences in the behavior of males and females, we ran the trap bioassay for each sex separately (using nine arenas per combination of spruce species and fungal isolate for each sex; 108 arenas in total). Throughout the 16-h test period, all arenas were kept in darkness at 20°C inside a cardboard box with holes to provide ventilation. Two large fans ensured continuous wind flow. After 16 h, the arenas were inspected for the number of beetles that had entered each trap, as well as any no-choice beetles that remained outside the traps on the filter paper.

### Statistical Analysis

All statistical tests were conducted in the R language using RStudio (RStudio, United States). Before analysis of continuous data (fungal lesion lengths, beetle entrance rates, and beetle tunnel lengths)was evaluated for normal distribution using the Shapiro-Wilk normality test. For normally distributed data, one-way ANOVA was performed using the “aov” function, followed by Tukey’s HSD to test for significant differences between individual treatments. For continuous data that were not normally distributed, the non-parametric Kruskal-Wallis with Dunn’s *post hoc* test was applied using the “kwAllPairsDunnTest” function. Beetle choice data from the single spruce species and trap bioassays were analyzed with Wilcoxon signed ranked test using the “wilcox.test” function. The “ggplot2” library was used to visualize data and make figures. Technical replicates from the multi- and single-species host preference bioassays were averaged before statistical analysis. The arenas made with bark from each spruce bolt (i.e., tree individual) in these bioassays were not true biological replicates, but rather technical replicates, since individual trees were the unit of replication.

## Results

### Fungal Pathogenicity

**Figure 4:**
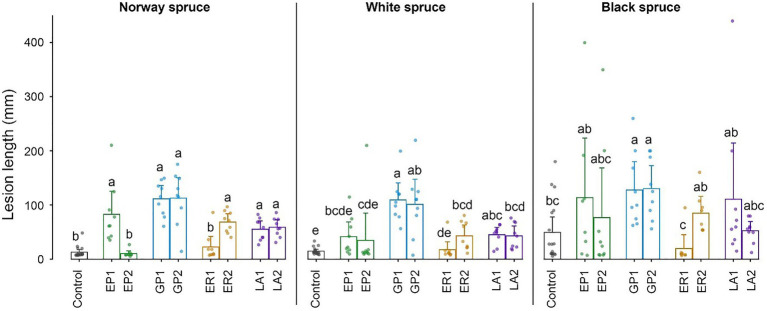
Lengths of necrotic lesions in the inner bark of cut bolts of Norway spruce, white spruce, and black spruce 3 weeks after inoculation with four bark beetle-associated fungi. EP, *Endoconidiophora polonica*; GP, *Grosmannia penicillata*; ER, *Endoconidiophora rufipennis*; and LA, *Leptographium abietinum*. For each fungus, two isolates were inoculated (e.g., EP1 and EP2; see Table 1 for details). Agar alone (without fungus) was inoculated as a control. Bars show meanvalues with 95% CIs. Dots show individual replicates (*n* = 9). Treatments with different letters (a–e) differed significantly (ANOVA and Tukey’s HSD test with *p* < 0.05).

All the four tested fungal species were pathogenic to *I. typographus*’ historical host Norway spruce and produced significantly longer necrotic lesions in the bark than the mock control (Figure 4). One isolate of *E. polonica* and *E. rufipennis* were exceptions to this pattern and produced very small lesions. *Grosmannia penicillata* produced longer lesions in Norway spruce than the other fungi, but due to considerable variation between replicates it did not differ significantly from pathogenic isolates of the other three fungi. There were no significant differences in lesion lengths produced by conspecific fungi associated with *I. typographus* (*E. polonica* and *G. penicillata*) and allospecific fungi associated with *D. rufipennis* (*E. rufipennis* and *L. abietinum*). Overall, lesion lengths in the evolutionary naïve hosts white spruce and black spruce were similar to those in Norway spruce. However, due to much variation between replicates in black spruce the only fungal isolates that differed significantly from the mock control in this spruce species were the two *G. penicillata* isolates (Figure 4). These isolates did not differ significantly from the other inoculated isolates (except for the non-pathogenic *E. rufipennis* isolate). In white spruce, *G. penicillata*, *L. abietinum*, and *E. rufipennis* (one isolate) produced significantly longer lesions than the mock control, whereas the two *E. polonica* isolates and one of the *E. rufipennis* isolates did not differ significantly from the control (Figure 4). The two *G. penicillata* isolates produced the longest lesions in white spruce, and one *G. penicillata* isolate produced significantly longer lesion than isolates from all other fungi, except one *L. abietinum* isolate.

### Host Choice Arenas With Multiple Spruce Species: Beetle Host Preferences

*Ips typographus* did not show any significant host preference when provided with a choice between Norway spruce, black spruce and white spruce in arenas with semi-natural bark media (Figure 1A). Beetles entered bark medium made from all three spruce species and there were no significant differences in entrance rate between the three species 18 h after the start of the bioassays (Figures 1B,C). However, only medium made from Norway spruce bark had a significantly higher entrance rate than the control medium (*p* = 0.02, Kruskal-Wallis with Dunn’s *post hoc* test). About one third of the beetles (32%) made no choice in the experiments and did not enter any medium. Beetles that entered the media tunneled 6.4 and 6.1 times longer in Norway spruce and black spruce medium than in control medium (*p* = 0.004 and *p* = 0.005, respectively; ANOVA and Tukey’s *post hoc* test). Tunneling length in white spruce was intermediate between that in Norway/black spruce and the control and did not differ significantly from either (Figures 1D,E). After 3–4 days, we observed fungal growth on all types of media, probably due to contamination by fungi carried into the arenas by the beetles.

### Choice Arenas With Single Spruce Species: Beetle Host Preferences in Fungus-Colonized Bark Medium

Beetles consistently preferred to enter medium colonized by fungus when given a choice between colonized and un-colonized spruce bark medium (Figures 2A,B). For all the 12 tested combinations of spruce species and fungal species significantly more beetles entered fungus-colonized medium than un-colonized medium (*p* < 0.05, Wilcoxon rank-sum test with continuity correction; Figure 2C). There were no clear differences in beetle preferences between spruce or fungal species. Out of 324 beetles used in the single-species bioassays, only four individuals entered un-colonized bark medium, 211 entered medium colonized by fungus, and 109 made no choice and remained on the surface of the media. There were more no-choice beetles in arenas with black spruce compared with Norway spruce and white spruce (49 vs. 32 and 28 beetles, respectively) and twice as many no-choice beetles in arenas with *E. polonica* colonization (39 beetles) compared with arenas with *E. rufipennis* colonization (19 beetles; Figure 2C).

**Figure 5:**
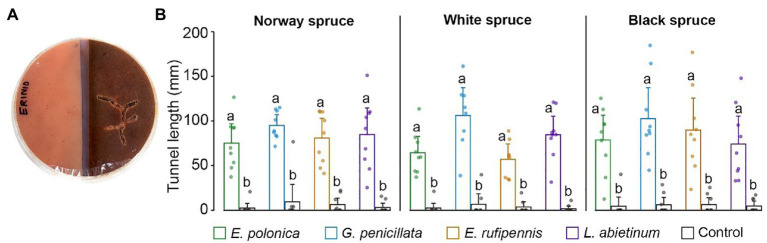
**(A)** Bark beetle tunneling in single-species choice arenas containing semi-circles of bark agar medium made from Norway spruce, black spruce, or white spruce. The medium was either colonized by one of four fungal species 15 days earlier (right) or was un-colonized (left). **(B)** Beetle tunneling length in un-colonized bark agar (Control) and in bark agar colonized by different bluestain fungi 5 days after the beetles were introduced into the arenas. Bars show meanvalues with 95% CIs. Dots show individual replicates (*n* = 9 individual trees per spruce species). Treatments with different letters (a, b) differed significantly (ANOVA and Tukey’s HSD test with *p* < 0.05). E, *Endoconidiophora*; G, *Grosmannia*; and L, *Leptographium*.

Beetles always tunneled significantly more in fungus-colonized spruce bark medium than in un-colonized medium (*p* < 0.05; ANOVA, Tukey’s *post hoc* test; Figures 5A,B). This was true for all combinations of spruce species and fungal species, with on average 10.1–48.4-fold more tunneling in fungus-colonized compared to un-colonized bark medium for the different spruce species-fungus species combinations. Beetles tunneled most in media colonized by *G. penicillata* (mean tunnel length 101.2 mm across all spruce species), but tunnel length in media colonized by this fungus was not significant longer than in media colonized by the other three fungi (76.7, 77.5, and 78.7 mm for *E. polonica*, *E. rufipennis*, and *L. abietinum*, respectively).

### Trap Bioassays: Beetle Attraction to Fungal Volatiles

Beetles actively selected fungus-colonized spruce bark medium based on olfaction. When given a choice between fungus-colonized and un-colonized medium in trap bioassays most beetles entered traps containing spruce bark medium colonized by fungi (Figures 3A,B). There were no differences in the behavior of males and females (data not shown), so data for both sexes were pooled. Out of 432 tested beetles, 277 (64%) entered traps with fungus, 103 (24%) entered traps with un-colonized medium, and 52 (12%) did not enter any traps (Figure 3B). Not a single beetle entered the empty traps. Out of 12 tested combinations of fungi and spruce species, 11 combinations had significantly more beetles in traps with fungus-colonized medium than in traps with un-colonized control medium (*p* < 0.05, Wilcoxon signed-rank test with continuity correction; Figure 3B). The only non-significant combination was white spruce medium colonized by *E. rufipennis*. The beetles showed no clear preferences for any specific fungal species or spruce species, but more beetles entered traps with media colonized by *G. penicillata* (85 beetles) than other fungi (61–68 beetles) and slightly fewer beetles entered traps with fungus-colonized white spruce medium (all fungal species; 86 beetles) compared with Norway spruce (98 beetles) and black spruce medium (93 beetles; Figure 3B).

## Discussion

Global trade leads to introduction of bark- and wood-boring beetles and other invasive pests worldwide and contribute to large-scale degradation of ecosystems (Wingfield et al., 2001, 2017; Paini et al., 2016). The European spruce bark beetle, *I. typographus*, is the most damaging pest of mature Norway spruce trees in Europe and is currently killing millions of spruce trees (Biedermann et al., 2019). Previous studies have shown that this European species can successfully colonize and breed in evolutionary naïve North American spruce species such as Sitka spruce [*Picea sitchensis* (Bongard) Carrière], white spruce (*P. glauca*) and black spruce (*P. mariana*; Økland et al., 2011; Flø et al., 2018). Thus, *I. typographus* could pose a significant threat to spruce species in North America if it becomes established there. Although *I. typographus* may be exposed to North American spruce species that have been planted in Europe the beetle has probably not adapted to colonize these exotic spruces. Any contact between these species started relatively recently and only takes place in restricted geographical areas, as North American spruce species tend to be planted in coastal regions in the British Isles, France, Denmark, and Norway, outside the geographical range of *I. typographus* (Mason and Perks, 2011).

Successful attacks by *I. typographus* and other tree-killing bark beetles are, in part, due to their intricate relationships with ophiostomatoid fungi in the genera *Endoconidiophora*, *Grosmannia*, and *Ophiostoma*, which help beetles to colonize trees and complete their development in the bark (Kirisits, 2007; Six and Wingfield, 2011). Unlike some bark beetle species, *I. typographus* seems to have dynamic interactions with ophiostomatoid fungi, vectoring assemblages of fungal species that may vary in time and space (Kirisits, 2007; Linnakoski et al., 2012, 2016). In this study, we have shown that several fungi vectored by *I. typographus* or the North American spruce beetle *D. rufipennis* are pathogenic to both North American spruce species and to Norway spruce, the historical host of *I. typographus*. Furthermore, we found that *I. typographus* was strongly attracted to medium colonized by all tested fungi and did not discriminate between its conspecific fungal associates and allospecific fungi vectored by *D. rufipennis*. Finally, we show that fungal colonization increased beetle tunneling into spruce bark medium irrespective of the geographical origin of the fungi. Collectively, these results suggest that *I. typographus* may colonize North American spruce species and form associations with native fungi that may increase the beetles’ colonization success in novel spruce hosts. Because the allospecific fungi *E. rufipennis* and *L. abietinum* only occur in North America there has probably been no previous contact between *I. typographus* and these fungi.

Symbiotic fungi associated with invasive bark and ambrosia beetles may promote successful invasion of their vector by helping it overcome the resistance of evolutionary naïve host trees. The invasive success of the pine-infesting red turpentine bark beetle *Dendroctonus valens* LeConte and its main fungal associate *Leptographium procerum* (W.B. Kendr.) M. J. Wingf. in China was mainly due to evolution of a more virulent *L. procerum* genotype following entry into China (Lu et al., 2011; Sun et al., 2013; Taerum et al., 2013). Similarly, the red bay ambrosia beetle, *Xyleborus glabratus* Eichhoff, has a mutualistic relationship with a pathogenic fungal symbiont, *Raffaelea lauricola* T.C. Harr., Fraedrich & Aghayeva, the causative agent of laurel wilt disease. In its invasive range in south-eastern United States this ambrosia beetle-fungus complex attacks healthy trees, but in their native range in Asia the beetle and the fungus are not known to damage their host trees (Koch and Smith, 2008; Hulcr and Dunn, 2011).

The ability of bark beetle-associated fungi to detoxify conifer chemical defenses is probably critical for *I. typographus* and other species that colonize live trees (Berryman, 1972; Paine et al., 1997; Franceschi et al., 2005; Lieutier et al., 2009; Krokene, 2015). Successful fungal colonization of phloem, cambium and sapwood exhausts tree defenses and prevents trees from mounting induced defenses that could kill adult beetles and developing larvae (Krokene, 2015). Attacked trees start to form necrotic lesions impregnated with terpenoids and phenolics around beetle entrance holes in an attempt to compartmentalize the damage and contain the attackers (Franceschi et al., 2005). Larger lesions are formed in response to more virulent fungi that require stronger defense responses to be contained (Krokene and Solheim, 1999). However, several fungal associates of *I. typographus*, such as *G. penicillata* and *E. polonica*, can exhaust tree defenses by inducing synthesis of terpenoids and phenolics in infected tissues and then actively metabolize these chemical defenses (Hammerbacher et al., 2013, 2019; Wadke et al., 2016; Zhao et al., 2019). For example, despite rapid activation of genes involved in phenolic synthesis in Norway spruce bark infected by *E. polonica*, phenolics concentrations in the bark drop because the fungus metabolizes phenolics and use them for their nutrition (Hammerbacher et al., 2013).

Our results showed that fungi associated with *I. typographus* or *D. rufipennis* had comparable pathogenicity in historical and naïve spruce hosts. *Grosmannia penicillata*, a common symbiont of *I. typographus*, grew well in both Norway spruce, black spruce, and white spruce and caused longer lesions than most other fungi in all three spruce species. *Grosmannia penicillata* has been found associated with different bark beetle species in Europe and may be well adapted to colonize different spruce and pine species (Kirisits, 2007; Linnakoski et al., 2012). Additionally, *G. penicillata* metabolizes phenolics more effectively than other fungal associates of *I. typographus* (Zhao et al., 2019). The other con- and allospecific fungi in our study induced comparable lesion lengths and were at least moderately virulent to all inoculated spruce species. Thus, all the fungi tested in this study could probably promote successful host colonization by *I. typographus* in novel spruce hosts.

Volatiles may mediate interactions between insect vectors and mutualistic microbial symbionts, as reported in ambrosia beetles that are attracted to nutritionally beneficial fungal symbionts through olfaction and avoid non-symbiotic fungi (Hulcr et al., 2011). Fungal volatiles might play an important role in host finding by bark beetles searching for suitable breeding and feeding sites in novel habitats with naïve host trees. Fungal volatiles may also be used as cues by beetles inside the bark of the host tree. Newly eclosed *I. typographus* adults seem to recognize and seek out fungal symbionts during the maturation feeding phase before they disperse from the brood tree, and they seem to mainly use volatile compounds emitted by the fungi (Kandasamy et al., 2019). After eclosion from pupa, the immature callow adults feed intensively on fungus-colonized inner bark until they acquire enough energy for dispersal and sexual maturation. Specific olfactory sensory neurons on the beetles’ antennae detect aliphatic and aromatic alcohols and their esters, such as 2-phenyethanol, 2-phenylethyl acetate, 3-methyl-1-butanol, 3-methylbutyl acetate, and other volatiles produced by *E. polonica* and *G. penicillata* (Kandasamy et al., 2019). An artificial blend containing biologically relevant concentrations of these compounds attracted newly eclosed *I. typographus* adults in an olfactometer assay (Kandasamy et al., 2019).

The qualitative and quantitative composition of the volatile blends produced by ophiostomatoid fungi, and thus the effect they have on bark beetle behavior, probably varies between fungal species and the spruce species they colonize. Therefore, we expected *I. typographus* to be most attracted to conspecific fungi growing on their historical spruce host. But contrary to our expectations, *I. typographus* was strongly attracted to volatiles emitted by both con- and allospecific fungi, and we found no clear effect of which spruce species the fungi were colonizing. This may suggest that con- and allospecific fungi produce similar volatile profiles that were little influenced by the spruce bark medium they were colonizing. Closely related ophiostomatoid fungi have been found to produce similar volatile profiles and this could explain why *I. typographus* is attracted to fungal species it has no previous experience with Kandasamy et al. (2016, 2019). The conspecific fungi *E. polonica* and *G. penicillata* produce 2-phenylethanol, 2-phenylethyl acetate, 3-mehtyl-1-butanol, and 3-methyl-1-butyl acetate that are attractive to *I. typographus* (Kandasamy et al., 2019). Interestingly, the allospecific fungus *L. abietinum* also produces some of these behaviorally active compounds (Kandasamy et al., 2016). The volatile profile produced by *E. rufipennis* is unknown, but since it is taxonomically close to *E. polonica* this species might be expected to produce a similar volatile profile (de Beer et al., 2014).

The fact that beetles were attracted to fungi growing on all three spruce media indicates that the chemical composition of the naïve spruce trees had little impact on the production of attractive cues by fungi. All the fungi in our study grew well on bark medium from naïve hosts and were as attractive to the beetles on naïve host medium as when they were colonizing their native spruce host. The chemical constituents of Norway spruce bark are well-studied, whereas the bark chemistry of black and white spruce is less well known. However, a comparative study of Norway spruce and two other North American spruce species showed very similar terpene profiles across all three species (Flø et al., 2018). In our trap bioassays, we found that *I. typographus* also showed some preference for spruce bark medium without fungal colonization. About one third of the tested beetles entered traps with un-colonized bark medium made from Norway spruce, black spruce, or white spruce, but not a single beetle entered empty traps without medium. Thus, *I. typographus* seems to be attracted also to volatiles originating from un-colonized bark of both historical and novel spruce hosts.

Bark beetles use host volatiles and pheromones as important long-range cues for recognizing suitable host trees (Zhang and Schlyter, 2004; Raffa et al., 2016). However, the decision to accept or reject a particular tree depends on short-range olfactory, tactile, and gustatory cues. Non-volatile phenolic compounds have been suggested to play key roles in host acceptance (Faccoli and Schlyter, 2007; Huang et al., 2020; Netherer et al., 2021). In the absence of fungal colonization, *I. typographus* did not distinguish between different spruce species in our multi-species bioassays and entered medium made from all three spruce species to the same degree. Additionally, beetles tunneled similar lengths in bark medium made from historical and naïve spruce hosts. Økland et al. (2011) also found no significant differences between historical and novel spruce hosts in several beetle performance traits such as gallery length, number of offspring produced and offspring weight. This suggests that important bark constituents are similar between spruce species and that many host trees provide an adequate medium for brood development. Colonization by fungus increased beetle preference and tunneling in all three bark media irrespective of fungal species. Previously, *I. typographus* was found to avoid a medium infused with catechin, probably due to the anti-feedant and anti-nutritional properties of this phenolic compound (Hammerbacher et al., 2019). The strong beetle tunneling into media colonized by the allospecific fungi *L. abietinum* and *E. rufipennis* suggests that these fungi fulfill similar detoxification roles as *E. polonica* and *G. penicillata* and therefore could promote *I. typographus* host acceptance in naive spruce species.

Strong attraction and preference of beetles to tunnel in fungus-colonized medium indicates the importance of fungal symbionts to *I. typographus*. Fungal symbionts play multiple roles in bark beetle colonization and development by overwhelming tree defenses, regulating attack densities, metabolizing host defenses, and translocating and providing essential nutrients (Schlyter et al., 1987; Lieutier et al., 2009; Wang et al., 2014; Wadke et al., 2016; Six and Elser, 2019; Netherer et al., 2021). Larvae or immature adults of many bark beetles can fulfill their nutritional requirements by feeding on fungi alone or on fungus-colonized phloem (Six, 2012, 2013). Our study further confirms that fungal volatiles play a crucial role for bark beetles in locating host substrates colonized by beneficial fungi. Furthermore, by feeding on fungus-colonized tissues the beetles obtain additional nutrients provisioned by the fungi and reduced concentrations of tree defense compounds due to fungal metabolism. The fungi, on their side, increase their dispersal rate by attracting beetle vectors that transport fungal spores to new host trees.

## Conclusion

*Endoconidiophora polonica* and *G. penicillata* are considered to be the most virulent fungi associated with *I. typographus* in Europe. Using these two species and the allospecific fungi *E. rufipennis* and *L. abietinum*, we showed that *I. typographus* does not rely on specific fungal species to colonize different spruce species. Based on our *in vitro* assays, we predict that if *I. typographus* is introduced in North America it could colonize evolutionary naïve spruce trees with support from conspecific fungi. In addition, *I. typographus* could form novel associations with allospecific fungi. However, the outcome of direct and indirect interactions between con- and allospecific fungi competing for shared resource in the phloem and sapwood is not known. Competitive interactions among fungi are mediated by several factors such as temperature, bark moisture content, and tree chemistry (Solheim, 1991; Hofstetter et al., 2005; Dysthe et al., 2015; Wang et al., 2020). Still, *I. typographus* seems to have a dynamic relationship with fungal symbionts in its historical range and this symbiont plasticity could preadapt this beetle to establish novel relationships with allospecific fungi that could increase beetle fitness in naïve habitats.

## Data Availability Statement

The raw data supporting the conclusions of this article will be made available by the authors, without undue reservation.

## Author Contributions

PK and ST planned the study with input from DK and analyzed the results. ST did the field and lab work. ST, PK, and DK wrote the paper. All authors contributed to the article and approved the submitted version.

### Conflict of Interest

The authors declare that the research was conducted in the absence of any commercial or financial relationships that could be construed as a potential conflict of interest.
